# Haploinsufficiency predictions without study bias

**DOI:** 10.1093/nar/gkv474

**Published:** 2015-05-22

**Authors:** Julia Steinberg, Frantisek Honti, Stephen Meader, Caleb Webber

**Affiliations:** 1 MRC Functional Genomics Unit, Department of Physiology, Anatomy and Genetics, University of Oxford, Oxford OX1 3PT, UK; 2 The Wellcome Trust Centre for Human Genetics, University of Oxford, Oxford OX3 7BN, UK

## Abstract

Any given human individual carries multiple genetic variants that disrupt protein-coding genes, through structural variation, as well as nucleotide variants and indels. Predicting the phenotypic consequences of a gene disruption remains a significant challenge. Current approaches employ information from a range of biological networks to predict which human genes are haploinsufficient (meaning two copies are required for normal function) or essential (meaning at least one copy is required for viability). Using recently available study gene sets, we show that these approaches are strongly biased towards providing accurate predictions for well-studied genes. By contrast, we derive a haploinsufficiency score from a combination of unbiased large-scale high-throughput datasets, including gene co-expression and genetic variation in over 6000 human exomes. Our approach provides a haploinsufficiency prediction for over twice as many genes currently unassociated with papers listed in Pubmed as three commonly-used approaches, and outperforms these approaches for predicting haploinsufficiency for less-studied genes. We also show that fine-tuning the predictor on a set of well-studied ‘gold standard’ haploinsufficient genes does not improve the prediction for less-studied genes. This new score can readily be used to prioritize gene disruptions resulting from any genetic variant, including copy number variants, indels and single-nucleotide variants.

## INTRODUCTION

The cost of sequencing has decreased sharply in the last few years, making it possible to examine the genetic contribution to disease encoded within the exomes of tens of thousands of patients. However, as apparently healthy individuals also possess multiple genetic variants that disrupt protein-coding genes (1,2), distinguishing those loss-of-function (LoF) variants that influence the phenotype of a given patient from those that do not remains a significant challenge. Nonetheless, this prediction remains a crucial bottleneck in a variety of applications, such as identifying disease-causing *de novo* variants, or assessing mutational loading onto genes or biological pathways in case-control studies.

Approaches to predict the systemic or organismal effect of individual protein-coding gene disruptions frequently make use of existing information regarding gene function (3,4). However, genes in the genome have been studied very unevenly (Supplementary Figure S1). Consequently, the information used in a particular method is often available only for a subset of genes. For example, Gene Ontology (5) is one of the largest databases with functional annotations for genes; nonetheless, high-quality Gene Ontology gene annotations based on experimental data or trusted author statements are currently only available for less than 14 000 genes. Unfortunately, it is inevitably the less-studied genes that are of particular interest when making predictions. By contrast, the sets of haploinsufficient (HIS) genes used to both train and test different methods consist predominantly of very well-studied genes.

Here, we show that the study biases inherent in many biological networks affect the ability of existing methods to predict how likely each protein-coding gene is to be HIS (3) (meaning that two gene copies are needed to maintain normal function) or essential (4) (meaning that at least one gene copy is needed for normal function). Consequently, we devise a haploinsufficiency score for genes by integrating large-scale data without study bias, such as gene co-expression networks and a novel score derived from exonic variation in over 6000 individuals. Taking advantage of recently available less-biased gene sets for evaluation, we compare this method to previously published methods (3,4,6). We show that the new score characterizes a higher number of genes that are not well represented within published studies and performs significantly better on available sets of such less-studied genes, thereby providing a less-biased approach for this critical step in disease genomics.

## MATERIALS AND METHODS

A list of 23 019 human protein-coding genes with Ensembl gene IDs was downloaded from Ensembl 70.

Two hundred ninety-seven known HIS genes were taken from a paper by Dang *et al*. (7). A list of haplosufficient (HS) genes was compiled as 3794 human genes which are disrupted by deletion CNVs in healthy individuals (8). In particular, a gene was defined as disrupted if a CNV overlapped an exon in all transcripts of the gene.

### Huang *et al*. data

We obtained predicted haploinsufficiency probabilities from a paper by Huang *et al*. (3), considering the predictions for 17 082 genes based on imputation of missing information; we refer to his score as ‘Huang HIS score’. Converting gene symbols to Ensembl gene IDs, we retained predictions for 17 069 genes. Moreover, we obtained the HumanNet v1 integrated functional linkage network from http://www.functionalnet.org/humannet/. After conversion of Entrez gene IDs to Ensembl gene IDs, we retained 15 827 unique genes and 469 383 links. For each gene, we calculated the number of links in the networks, and the sum of link weights to 302 known HIS genes (7,9) as done by Huang *et al*. (3).

### Khurana *et al*. data

We obtained the predicted indispensability score from a paper by Khurana *et al*. (4) for 21 863 human genes, which we refer to as ‘Essentiality score’. Converting gene symbols to Ensembl gene IDs yielded scores for 18 386 genes. Moreover, for each of these genes, the authors provided the number of links (or gene degree) in various networks, namely in protein−protein interaction (PPI), metabolic, genetic interaction, phosphorylation, regulatory and signalling networks, as well as in the integrated network ‘Multinet’ (4). For each gene, the authors also provided the number of these networks that the gene was part of, as well as a ‘disease significance score’ (three for known essential genes, two for genes with disease annotations in HGMD, one for LoF tolerant genes and zero for all other genes) (4).

### Petrovski *et al*. data

We obtained the Residual Variance Intolerance Score (RVIS) from a paper by Petrovski *et al*. (6) for 16 956 human genes. Converting gene symbols to Ensembl gene IDs, we retained the RVIS for 16 572 human genes.

### Pubmed papers

For each human gene, the list of Pubmed papers citing that gene was obtained from Pubmed on 20 May 2014. Pubmed gene IDs were converted to Ensembl gene IDs using the conversion file supplied by Pubmed. We then calculated the total number of Pubmed papers for each gene.

### Gene coding-sequence length

The coding-sequence (CDS) length of 22 878 human protein-coding genes with Ensembl gene IDs were downloaded from Ensembl 72. The CDS of each gene was set to the CDS of the longest transcript.

### Co-expression networks

We downloaded the COEXPRESdb human gene co-expression network v13.1 on 03/03/2014. Entrez gene IDs were converted to Ensembl gene IDs. Moreover, we only considered gene links with correlation *r* ≥ 0.3, yielding 3 566 815 unique links between 15 277 genes.

We downloaded the GTEx Pilot 1 data on 29 April 2013. We excluded genes with RPKM < 1 in >95% of the samples and calculated gene co-expression using weighted Pearson correlation as in COEXPRESdb [http://coxpresdb.jp/help/coex_cal.shtml, 3 March 2014]. We only considered gene links with correlation *r* ≥ 0.3, yielding 23 278 495 unique links between 15 949 genes.

For each human gene, we obtained the distance to known HIS genes in a co-expression network as the sum of the 20 highest links weights to the 297 known HIS genes. The choice of this threshold did not strongly influence the results as the distances were highly correlated with distances from the 10, 30 highest or all links (Spearman ρ > 0.99 for COEXPRESdb, ρ > 0.88 for GTEx).

### NoVaDs

We downloaded human gene variation data from over 6000 exomes from the NHLBI exome server on 8 April 2014. Hugo gene symbols were converted to Ensembl gene IDs; we only considered variants that passed original QC filters. Similar to Petrovski *et al*. (6), we defined ‘rare’ variants as those with minor allele frequency (MAF) ≤ 0.1% combined in all samples, and ‘common’ variants as those with MAF > 0.1%. Non-synonymous variants were defined as variants annotated by the terms ‘missense’, ‘missense-near-splice’, ‘splice-3’, ‘splice-5’, ‘stop-gained’, ‘stop-gained-near-splice’, ‘stop-lost’ and ‘stop-lost-near-splice’. Synonymous variants were defined as variants annotated by ‘coding-synonymous’ or ‘coding-synonymous-near-splice’.

We obtained the Petrovski *et al*. ‘RVIS’ (6) for genes as the studentized residuals from regressing the number of common non-synonymous variants on the total number of variants in genes.

We also obtained a Non-synonymous Variation Depletion score (‘NoVaDs’) as the ratio of the number of common to the number of rare non-synonymous variants in each gene. Notably, the NoVaDs is strongly correlated with the RVIS (Spearman ρ = 0.77, *p* < 10^−100^), but not affected by different codon usage between genes.

We also examined how the NoVaDs was affected by the chosen cut-off of MAF > 0.1% for common variants. To this end, we considered the alternative with cut-off MAF > 1% for common variants (denoted ‘NoVaDs_1%’) and the alternative with cut-off MAF > 0.01% for common variants (denoted ‘NoVaDs_0.01%’). Both the NoVaDs_1% and the NoVaDs_0.01% were highly correlated with the NoVaDs (Spearman ρ = 0.69 and ρ = 0.62, respectively) and were significantly worse than the NoVaDs at distinguishing disease genes (see Supplementary Data). Consequently, we proceeded with the NoVaDs defined with MAF > 0.1% for common variants.

### Evolutionary constraint

To calculate evolutionary constraint *dN*/*dS*, we downloaded *dN* and *dS* values for 16 511 human genes with one-to-one orthologues in macaque from Ensembl 62. If either *dN* or *dS* was 0, the value was set to 0.001 (1057 genes for *dN*, 35 genes for *dS*).

### F2A gene expression

For human gene expression data, we used GNF's gene atlas for the MAS5-condensed human U133A and GNF1H chips (10). Expression levels were mapped to LocusLink identifiers and to 17 226 Ensembl genes using the annotation tables supplied by GNF. For each gene, the ratio of gene expression in foetal to adult tissue (referred to as ‘F2A expression ratio’) was derived by dividing the median expression level in four foetal tissues (Supplementary Table S1) by the median expression level in 31 adult tissues (Supplementary Table S1).

### Study bias

**Figure 1.**
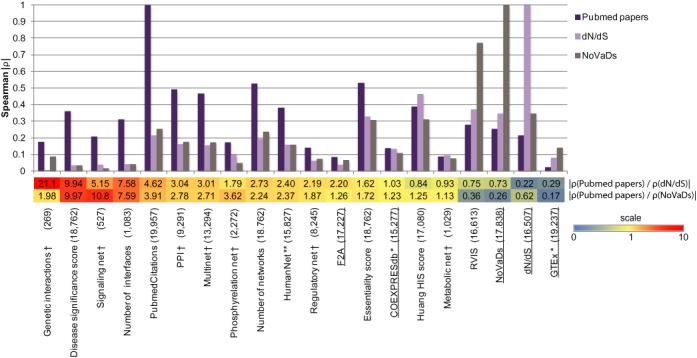
The presence and gene degree in biological networks is strongly correlated with study bias. The bar plot shows the Spearman correlation of various indices with the number of Pubmed papers, evolutionary constraint *dN*/*dS* and the NoVaDs. Almost all correlations are significant (*p*-values see Supplementary Table S2). The lower panel shows the ratios obtained from Spearman correlation coefficients for each index, |*ρ*(Pubmed papers)/*ρ*(*dN*/*dS*)| and |*ρ*(Pubmed papers)/*ρ*(NoVaDs)|. Red colour highlights the indices with strong study bias (see colour scale). † = gene degree in network; ** = proximity to HIS genes from (7,9) as in (3); * = proximity to HIS genes from (7); Essentiality score = Khurana *et al*. (4) gene indispensability score; RVIS = Petrovski *et al*. (6) Residual Variance Intolerance Score; Huang HIS score = Huang *et al*. (3) haploinsufficiency probabilities. We underlined the datasets used to construct the genome-wide haploinsufficiency score (GHIS). For each index, the number in parentheses shows the number of genes with values for the index.

We calculated Spearman correlation coefficients of indices depicted in Figure 1 with the number of Pubmed papers for each gene; *dN*/*dS*; and the NoVaDs using R (see Supplementary Table S2).

### Construction of genome-wide haploinsufficiency score (GHIS)

A support vector machine (SVM) was used to construct a genome-wide haploinsufficiency score (GHIS) for human genes. We used the function ‘svmt’ in the e1071 library in R with options ‘decision.values = T’ and ‘probability = T’. Features were specified as distance to HIS genes in the COEXPRESdb co-expression network, distance to HIS genes in the GTEx co-expression network, the NoVaDs, *dN*/*dS* and F2A expression ratio. Missing values were replaced by 0 for the distance in co-expression networks, the median for the NoVaDs and *dN*/*dS*, and the mean for the F2A expression ratio.

We used a linear kernel SVM and performed 100 randomizations by sub-sampling 297 HS genes.

In each randomization, we used 10-fold cross-validation: we divided the 297 HIS, 297 HS genes into 90% training, 10% test set; fit an SVM to the training set and obtained GHIS predictions for the test set (using the ‘predict’ function in the e1071 library in R with option ‘decision.values = T’). This was repeated 30 times. We then averaged predicted values from the 30 repeats and evaluated the predictions using a Receiver Operator Characteristic (ROC) curve and the area under the curve (AUC) metric. The ROC curve is a plot of true positive versus false positive rate, while the AUC is the area under that curve. We obtained the AUC using the ‘trapz’ function in the R ‘ROCR’ library. To test the performance of this method, we used extensive randomizations and 10-fold cross-validation. When using 90% of the HIS and HS genes, the performance on the remaining 10% yielded a mean area under the ROC curve (AUC) of 0.67 (standard deviation 0.02) after excluding six randomizations with AUC < 0.5 (Supplementary Figure S8).

Subsequently, we fit an SVM to all 297 HIS, 297 HS genes and calculated the HIS score for all human genes using the ‘predict’ function in the e1071 library in R with options ‘decision.values = T’ and ‘probability = T’.

The final GHIS for each gene was obtained by averaging the predicted values for those 94 randomizations with AUC > 0.5 on the test set.

We checked that 100 randomizations were sufficient: the score obtained by repeating the process had Spearman correlation ρ > 0.996 with the GHIS. The predicted GHIS values are included in Supplementary Table S3.

We also considered using an SVM with radial kernel; the results were similar (see Supplementary Data).

### Known disease genes

We obtained the following tests lists from the paper by Petrovski *et al*. (6): 175 genes annotated as HIS in OMIM (‘OMIM HI’); 108 genes annotated as HIS with known *de novo* mutations in OMIM (‘OMIM HI *de novo*’).

Moreover, we obtained a list of 818 genes annotated as autosomal dominant (AD) disease genes in the Clinical Genomics Database (CGD) on 5 June 2014. After conversion of gene symbols to Ensembl gene IDs, 803 ‘CGD AD’ genes remained. While some of these genes might cause disease through gain of function due to the observed variants, this information was not available.

In addition, we also considered the consequences of gene disruptions of human orthologues in the mouse given the broad conservation of associated characteristics (11). We obtained the phenotypes exhibited by mouse models possessing a targeted heterozygous disruption of a protein-coding gene from Mouse Genomics Informatics (MGI), downloaded on 18 April 2014.

Similarly to Petrovski *et al*. (6), we considered genes for which heterozygous disruption yields embryonic, pre- or perinatal lethality phenotypes (‘MGI Lethality’), and a set of genes for which heterozygous disruption yields seizures (‘MGI Seizures’). Using human-mouse gene one-to-one orthologue information, we mapped these genes to 146 human ‘MGI Lethality’ genes and 56 human ‘MGI Seizures’ genes.

We also downloaded a list of mouse genes for which heterozygous disruption of the gene yielded significantly reduced viability at weaning from the Sanger Mouse Resources Portal on 7 April 2014. Using human-mouse gene one-to-one orthologue information, we mapped these genes to 311 human genes (‘SMP Viability’).

Finally, we obtained a list of all human genes whose one-to-one mouse orthologue when disrupted yields an abnormal mouse phenotype from the MGI database on 10 December 2012. We obtained a subset of the ‘SMP Viability’ genes not contained in the list downloaded from MGI, and refer to this list as the ‘SMP Viability new’ genes.

After removal of the genes we used for training the SVM as well as the genes Khurana *et al*. (4) used for training, we retained 55 ‘OMIM HI’ genes; 32 ‘OMIM HI *de novo*’ genes; 550 ‘CGD AD’ genes; 88 ‘MGI Lethality’ genes; 37 ‘MGI Seizures’ genes; 198 ‘SMP Viability’ genes; 124 ‘SMP Viability new’ genes.

### Disease candidate genes

We obtained the following lists of disease candidate genes: 59 genes disrupted by *de novo* LoF mutations in autism probands (12) (‘ASD1’); 65 genes disrupted by *de novo* LoF mutations in other sets of autism probands (13–15) (‘ASD2’); 122 genes as the union of ASD1 and ASD2 (‘ASD12’). After removal of the genes we used for training the SVM as well as the genes Khurana *et al*. (4) used for training, we retained 50 ‘ASD1’ genes, 49 ‘ASD2’ genes and 98 ‘ASD12’ genes.

We also considered 18 genes disrupted by *de novo* LoF mutations in at least two autism probands from a larger study (16) (which included the probands from (12) and (13–15)). This set, referred to as ‘ASD_M’ was only used as showcase for practical application, so the training genes were not excluded (the conclusions from results for 12 genes not in the training sets remain unchanged throughout).

### Comparison of predictions

For each of the seven lists of known disease genes and for each of the three lists of disease candidate genes, we compared the predictions from three previously published scores (‘Huang HIS score’ (3), ‘Essentiality score’ (4), RVIS (6)) to the GHIS. We used the AUC and Matthew's Correlation Coefficient (MCC) metrics. For each set of predictions and each gene list, we compared the gene list to 100 random lists with the same number of genes, matching genes for CDS length. The matching for CDS length was done by taking each gene on the list and substituting it with one of 100 genes with the closest CDS length. When considering gene sets based on human-mouse one-to-one orthologues, we considered random gene sets chosen from 15 765 human genes with one-to-one human-mouse orthologues only, again matching for CDS length.

The MCC was obtained defining the 25% of genes with the highest deleteriousness score from a method as ‘predicted HIS’, all other genes as ‘predicted HS’ genes. For each list of known or candidate disease genes and a list of matched random genes, the MCC is then equal to $\frac{{\rm TP \times TN - FP \times FN}}{{\sqrt {(\rm TP + FP)(\rm TP + FN)(\rm TN + FP)(\rm TN + FN)} }}$, where TP means ‘predicted HIS’ disease genes, TN means ‘predicted HS’ random genes, FP means ‘predicted HIS’ random genes and FN means ‘predicted HS’ disease genes. For each gene list, we then compared the AUC and the MCC for the GHIS to the three other methods using the Mann–Whitney rank test in R.

For the ASD_M set of 18 autism candidiate genes as an example for a practical application, we evaluated how many fell into the top 1, 2, …, 99% percentile among the scored genes for the GHIS and the three other scores. Due to small numbers, this was descriptive only and no statistical analysis was performed.

## RESULTS

We sought a gene haploinsufficiency score that would not be influenced by how well-studied individual genes are. To construct this score, we first considered a range of biological datasets and existing predictions relevant to this aim.

### Study bias in biological methods and existing approaches

We first evaluated study bias in the gene-network approaches to predicting HIS genes employed by Khurana *et al*. (4) as well as that employed by Huang *et al*. (3) in detail. Khurana *et al*. employed six different types of networks and ‘Multinet’, a network integrating all of the six networks (4) (see Figure 1). For each gene, they calculated the number of connections in each network (the gene ‘degree’), and the number of networks that the gene was part of. These biological networks are partially constructed based on low-throughput experiments carried out only for genes of specific interest. However, the selection of ‘biologically interesting’ genes for small-scale experiments is known to impact which functional relationships (links) between genes are identified, and thus included in current representations of biological networks (17). We used the number of Pubmed papers associated with a gene as a measure of how well-studied genes are.

When we considered the extent to which study bias influences gene degree, we compared the correlation of a given score (such as gene degree) with the number of Pubmed papers per gene to the correlation of the score with human-macaque *dN*/*dS*, a measure of evolutionary conservation known to be higher for HIS genes (3) (Figure 1, Supplementary Table S2).

Indeed, we found a significant correlation with the number of Pubmed papers in each of the networks considered by Khurana *et al*. (Figure 1). Importantly, for all but the metabolic network, the gene degree has 1.8-fold to 21-fold higher correlation with number of Pubmed papers than with human-macaque *dN*/*dS*.

Similarly, the number of networks each gene participates in is 2.7-fold more strongly correlated with the number of Pubmed papers than with evolutionary conservation. Notably, the number of networks each gene participates in is the strongest predictor for the final score by Khurana *et al*. (‘Essentiality score’) and strongly correlated with this score (Spearman ρ = 0.85, *p* < 10^−100^). Consequently, the Essentiality score is 1.6-fold more strongly correlated with the number of Pubmed papers per gene than with evolutionary conservation.

In an alternative network-based approach, Huang *et al*. (3) considered the functional linkage network ‘HumanNet’ which integrates protein–protein interactions, gene co-expression, gene co-citation and other data to indicate how functionally similar pairs of genes are likely to be. The haploinsufficiency probability predicted by Huang *et al*. (‘Huang HIS score’) was largely based on the proximity of a query gene to known HIS genes in the HumanNet network (Spearman ρ = 0.59, *p* < 10^−100^). However, the proximity to known HIS genes in HumanNet also shows 2.4-fold stronger correlation with the number of Pubmed papers per gene than with evolutionary conservation (Supplementary Table S2).

**Figure 2.**
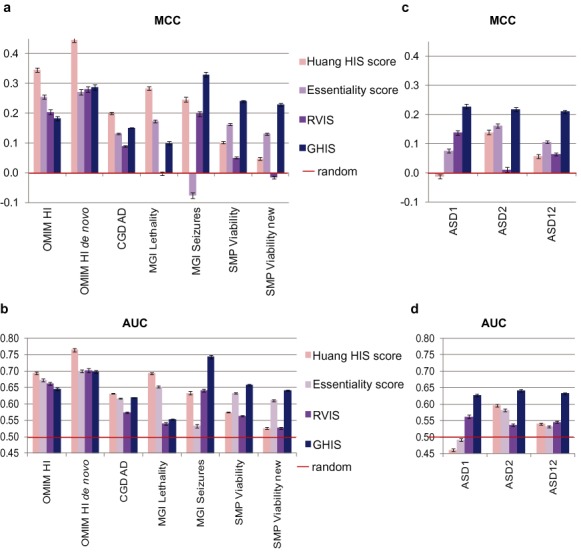
Comparison of four gene deleteriousness scores based on known disease genes and mouse models (Table 1) as well as candidate disease genes. (**a**) Comparison of scores based on known disease genes and mouse models using the MCC metric. (**b**) Comparison of scores based on known disease genes and mouse models using the AUC metric. (**c**) Comparison of gene scores based on candidate disease genes using the MCC metric. (**d**) Comparison of gene scores based on candidate disease genes using the AUC metric. The MCC takes values between −1 and 1, with higher values indicating better performance. The AUC gives the probability that a randomly chosen gene from the set has a higher score than a randomly chosen gene from the genome (accounting for length, see Materials and Methods section). Hence possible values lie between 0 and 1, with higher values indicating better performance. Each gene set was compared to random gene sets of equal size, accounting for coding-sequence length (see Materials and Methods section). The bar plots show mean values for 100 random comparison gene sets, error bars indicate standard errors. Mann–Whitney *p*- and *q*-values for comparison of scores are listed in Supplementary Tables S4 and S5.

To directly demonstrate the impact of the study bias on the performance of the scores for predicting HIS genes, full knowledge of the phenotypic consequences of gene disruption for all human genes would be required. As such data are currently not available, we compared the performance of the Essentiality score and the Huang haploinsufficiency score for predicting a set of well-studied HIS genes from OMIM (‘OMIM HI’; Table 1) and a set of less-studied human one-to-one orthologues of HIS mouse genes from the Sanger Mouse Project (‘SMP Viability’; Table 1). The OMIM HI genes have a significantly higher number of Pubmed papers per gene than the SMP Viability genes (Mann–Whitney *p* < 10^−8^; Supplementary Figure S2), with greater than 2-fold difference in the median. We used MCC and the area under the ROC curve (AUC) as performance metrics, comparing scores for genes in the study set to scores of random genes matched for CDS length (see **Methods**). Both the Essentiality score and the Huang haploinsufficiency score have significantly higher AUC and MCC for the OMIM HI set than the SMP Viability set (Figure 2a and c, Supplementary Tables S4 and S5). The better performance of these methods on the well-studied genes is consistent with the study bias inferred above.

**Table 1.** Gene sets used to evaluate the genome-wide haploinsufficiency score and three state-of-the-art approaches

Gene set	Description	Number of genes
OMIM HI	Online Mendelian Inheritance in Man (OMIM) haploinsufficient genes (as in (6))	55
OMIM HI *de novo*	OMIM haploinsufficient genes with *de novo* mutations listed in OMIM (as in (6))	32
CGD AD	Clinical Genomic Database (CGD) autosomal dominant disease genes	550
MGI Lethality	Human genes for which the heterozygous disruption of the one-to-one orthologue in mouse causes lethality (taken from Mouse Genome Informatics (MGI) database; analogous to (6))	88
MGI Seizures	Human genes for which the heterozygous disruption of the one-to-one orthologue in mouse causes seizures (taken from MGI; analogous to (6))	37
SMP Viablity	Genes for which the heterozygous disruption of the one-to-one orthologue in mouse yielded significantly reduced viability by weaning (taken from the Sanger Mouse Resources Portal (SMP))	198
SMP Viability new	SMP Viability genes without MGI phenotype records prior to 10 December 2012	124

### Large-scale datasets without study bias

To develop haploinsufficiency predictions less affected by study bias, we wished to consider large-scale biological datasets that were obtained from genome-wide data. Following a similar ethos, Petrovski *et al*. (6) proposed to measure whether each gene is depleted in common (MAF>0.1%) non-synonymous variation based on data from over 6000 exomes. They defined the RVIS as the studentized residual when the number of common non-synonymous variants was regressed on the total number of variants in each gene. However, we found that the derivation of the RVIS induced a strong correlation between gene CDS length and the absolute size of the RVIS (Pearson *r* = 0.5, *p* < 10^−100^; see **Methods**). Moreover, both the highest and lowest RVIS values were preferentially attained by the longest genes (Supplementary Figure S3a). This is largely due to the construction of the score, as we found similar dependence on CDS when randomizing the proportion of common non-synonymous variants among genes (see Supplementary Data, Supplementary Figure S4). Hence, the effects of CDS and intolerance to gene disruptions on the RVIS are difficult to disentangle. Moreover, the RVIS does not account for potential differences in the relative numbers of possible synonymous and non-synonymous mutations in genes.

Consequently, we instead derived an alternative score for the relative depletion of common functional variation in each gene: the ratio $\frac{{number\;of\;common\;nonsynonymous\;variants}}{{number\;of\;rare\;nonsynonymous\;variants}}$ (see **Methods**), which we call the ‘Non-synonymous Variation Depletion Score’ or NoVaDs. Intuitively, the intolerance of a population to functional variants in a given gene will act to decrease the MAF of such variants, thus decreasing the NoVaDs. The NoVaDs is not correlated with the CDS length of genes (*r* = −0.04, *p* < 10^−5^; Supplementary Figure S3b). Importantly, when gene length is accounted for, the NoVaDs distinguishes disease genes from random human genes better than the RVIS (Supplementary Figure S3c and d, Supplementary Table S6).

As expected, the NoVaDs is 1.36-fold more highly correlated with evolutionary conservation (Spearman ρ = 0.35, *p* < 10^−100^) than with Pubmed papers per gene (ρ = −0.26, *p* <10^−100^), thus not showing study bias.

As the correlation between NoVaDs and evolutionary conservation is only moderate, we also applied the NoVaDs to evaluate the biological networks considered by Khurana *et al*. (4) and Huang *et al*. (3) for study bias (Figure 1, Supplementary Table S2). The results showed evidence for study bias entirely consistent with the observations based on evolutionary conservation, hence providing additional evidence for bias in those networks.

By contrast, we found no evidence for study bias in large-scale gene co-expression networks (Figure 1, Supplementary Table S2), specifically COEXPRESdb (based on microarray data (18)) and a network constructed from the pilot 1 phase RNA-sequencing data of the Gene-Tissue Expression Consortium (GTEx (19)). Similarly to the approach of Huang *et al*. (3), we considered how strongly each gene is co-expressed with 297 known HIS genes (7) (see **Methods**). This value is strongly correlated with the gene degree in the networks (COEXPRESdb: ρ = 0.92; GTEx: ρ = 0.77; both *p* < 10^−10^), and does not show a study bias (Supplementary Table S2).

### Unbiased haploinsufficiency score predictions

To derive a score indicating how likely each human gene is to be HIS, we applied a machine learning method (an SVM; see **Methods**) to a range of gene features. Based on the results above, we used gene features that did not show study bias for the predictions, namely the co-expression with known HIS genes in the COEXPRESdb and GTEx co-expression networks; the NoVaDs; evolutionary conservation; and the ratio of gene expression in fetal to adult tissue (see **Methods**). As with other methods, to train the SVM we used HIS genes taken from a review (7) while HS genes were obtained as genes disrupted by deletion copy number variants in healthy individuals (8) (see **Methods**). HS genes were subsampled 100 times, averaging predicted HIS scores for each gene (see **Methods**). As our method is applicable to genes irrespective of their degree of study, we called the resulting score ‘Genome-wide haploinsufficiency score’ or GHIS.

We wanted to compare the GHIS to previously published methods, denoting the latter scores as ‘Essentiality score’ (4), RVIS (6) and ‘Huang HIS score’ (3) (see **Methods**). The GHIS provides a score for a higher number of genes than previously published methods (Table 2). Crucially, of the genes with a provided score, the GHIS includes about twice as many genes currently unassociated with any Pubmed papers as each of the three previously published methods, both in absolute numbers and as a proportion of the total predictions (Table 2).

**Table 2.** The genome-wide haploinsufficiency score evaluates a higher number of genes than three previously published methods, as well as a higher number of less-studied genes

Score	Total	Without Pubmed paper	Percent without Pubmed paper
GHIS	19 701	4621	23.46%
Huang HIS score	17 069	2064	12.09%
Essentiality score	18 386	1525	8.29%
RVIS	16 572	1774	10.70%

Essentiality score = Khurana *et al*. (4) gene indispensability score; RVIS = Petrovski *et al*. (6), Residual Variance Intolerance Score; Huang HIS score = Huang *et al*. (3) haploinsufficiency probabilities.

### Predictions for genes with disease association

In the next step, we evaluated the different scores on gene sets with known disease association (Table 1).

These genes have higher CDS length than general human genes (Supplementary Figure S5a). Consequently, we compared each gene set to 100 random gene sets with the same number of genes, matching genes for CDS length (see **Methods**). As in previous studies, MCC was used as primary comparison metric, assessing how many disease genes versus random genes fell into the genes predicted to be among the 25% most intolerant to disruption (cut-off chosen as used by Petrovski *et al*. (6); see **Methods**). We compared scores with the Mann-Whitney rank test.

The GHIS performed as well as or significantly better than the RVIS on all gene sets, and at least as well as the Essentiality score on all but the ‘OMIM HI’ and ‘MGI Lethality’ gene sets (all *q* < 10^−9^; Figure 2a, Supplementary Table S4).

The Huang HIS score outperformed the GHIS on the ‘OMIM HI’, ‘OMIM HI *de novo*’, ‘CGD AD’ and ‘MGI Lethality’ gene sets (all *q* < 10^−10^). However, these are some of the most studied human genes (median >50 papers/gene; Supplementary Figure S2a). By contrast, performance of the Huang HIS score declined steadily for less-studied genes, and the GHIS performed better than all published methods when predicting the considerably less-studied ‘SMP Viability’ and ‘SMP Viability new’ genes, as well as the ‘MGI Seizure’ genes (all *q* < 10^−10^).

The results were similar when considering the area under the ROC curve (AUC; see Figure 2b, Supplementary Table S5), although with better relative performance of the RVIS and Essentiality score. Notably, the GHIS again significantly outperformed all of the three other scores on the ‘SMP Viability’ and ‘SMP Viability new’ genes, as well as the ‘MGI Seizure’ genes (all *q* < 10^−10^).

### Predictions for disease candidate genes

Finally, we considered these methods’ predictions made for sets of disease candidate genes from recent exome sequencing studies. Autism probands have an elevated rate of *de novo* LoF mutations than unaffected individuals (20); around half of the *de novo* LoF mutations are expected to be causal (20), suggesting that the corresponding genes are HIS. As only about 50% of the ASD genes are likely to be causal, even with a score that distinguishes perfectly between HIS and HS genes, the MCC and AUC are expected to be lower than 1 for the ASD genes. Under the best-case scenario, we would expect the MCC to be around 0.38 and the AUC around 0.75 (see Supplementary Data).

We considered two independent sets of *de novo* LoF genes in autism (‘ASD1’, *n* = 50 (12); ‘ASD2’, *n* = 49 (13–15)) and their combination (‘ASD12’, *n* = 98). Genes with *de novo* mutations tend to have high CDS length (Supplementary Figure S5b), hence we accounted for CDS as for the disease gene sets above. As expected, the MCC and AUC for all four scores considered in this study are lower than under the best-case scenario (Figure 2c,d).

However, the GHIS significantly outperformed all three previously published scores on all three autism gene sets using the MCC metric (Mann–Whitney *q* < 10^−6^ for all three comparisons, Figure 2c, Supplementary Table S4), as well as using the AUC metric (Mann–Whitney *q* < 10^−10^ for all three comparisons; Figure 2d, Supplementary Table S5).

For the genes in the ASD1, ASD2 and ASD12 sets, we do not know which are causal, and therefore cannot evaluate the accuracy of the methods further. Consequently, as another example of practical application, we considered 18 genes disrupted by *de novo* LoF mutations in at least two autism probands from a larger study (16) (‘ASD_M’). All of these genes are associated with autism at <10% FDR based on *de novo* and transmitted genetic variants (16). Therefore, these genes should rank highly on a HIS score. We asked how many of the 18 ASD_M genes fell into the genes with the top 1, 2, …, 99% score for the GHIS and the three previously published scores. These genes have high CDS lengths (Supplementary Figure S6a), and their CDS is strongly correlated with their RVIS (Spearman ρ > 0.8) in agreement with the CDS length bias described above. Consequently, the results for the RVIS and the CDS are extremely similar (Supplementary Figure S6a) and it is difficult to quantify to which extend the ranking of these genes is confounded by mutations being more frequent in longer genes. While we accounted for the CDS-bias in the above analyses through randomizations, for this straightforward application, no like-for-like comparison of the RVIS to the three other methods was possible.

Of the remaining three scores, the GHIS performs at least as well as the Huang HIS score and the Essentiality Score across all possible cut-offs (Supplementary Figure S6b).

These results suggest that none of the methods considered in this study are accurate enough for use in a clinical setting, but that the GHIS has relatively the best performance on the best autism candidate genes.

## DISCUSSION

In this study, we have shown that the biological networks previously employed to predict haploinsufficiency are strongly impacted by study bias: well-studied genes are both part of more networks and have more links in individual networks. In particular, manual gene annotations based on low-throughput studies lead to both highly accurate, but also very biased networks (17,21). By contrast, systematic genome-wide assays aim to deliver information without study bias. Consequently, we used large-scale gene co-expression networks and information from thousands of exomes to construct a haploinsufficiency score (‘Genome-wide haploinsufficiency score’, GHIS) for over 19 700 human genes, of which over 23% are not at all well-studied. Subsequently, we showed that the GHIS outperforms previously published methods when assessing several disease gene sets that include less-studied genes.

While we found that the scores affected by study bias perform better on the well-studied genes considered here, this does not mean that such scores are preferable even on better-studied genes: if not accounted for, the bias could still lead to confounded and thus misleading results. By contrast, the GHIS is not affected by study bias (see also Supplementary Table S2), and is thus preferable to confounded scores.

There are limitations to our approach. Firstly, while we aimed to construct a haploinsufficiency score unaffected by study bias, the ‘gold-standard’ set of HIS genes used for training is very well-studied. Indeed, we found that a haploinsufficiency score constructed on unbiased data but tuned to the training set more strongly (see Supplementary Data) had a significantly better performance on well-studied genes, but a significantly worse performance for less-studied genes, and made predictions for fewer genes without Pubmed papers. Intuitively, fitting a predictor to capture known genes and thus their corresponding biological processes does not make the predictor more likely to successfully predict new genes with different biological mechanisms.

Secondly, even the less-studied test sets considered here have a median of over 30 Pubmed papers per gene, and are thus very well-studied compared to the majority of genes in the genome. Unfortunately, due to limited availability, we could not use mammalian HIS genes obtained from unbiased screens as training and test sets. Hence, we considered known human disease genes and genes whose one-to-one orthologue's disruption in the mouse indicates haploinsufficiency. However, most of the currently available models were constructed for well-studied human genes, and the information is limited to human genes with one-to-one orthologues in mice. Notably, the presence and severity of the phenotypes in mice might not transfer directly to humans (22).

Thirdly, our haploinsufficiency scores currently do not take the genetic background in individuals into account. This is a major limitation as genetic variants do not act in isolation: the genetic background has been shown to have an effect on animal models of gene disruption (23), and the disruption of multiple genes within the same biological pathway can increase the risk for a disorder (24). Higher-order models to predict the cumulative phenotypic impact of multiple genetic variants would require an extensive training set based on knowledge of the specific outcomes from various combinations of variants. A compromise might be to predict the phenotypic penetrance of gene-disruptive variants. The necessary data could become available from surveys of large numbers of well-phenotyped human individuals.

Finally, we note that none of the scores considered in this study had a very high performance (AUC>0.8) on any of the test sets. Hence, we would suggest that the GHIS could be used to test for enrichment of genes with high scores in a particular gene set of interest and/or to prioritize genes for further study; in the latter case, further scrutiny of individual gene predictions would be warranted.

In summary, we present an approach to predict haploinsufficiency for a broader range of human genes, without study biases inherent to previous methods.

## SUPPLEMENTARY DATA

Supplementary Data are available at NAR Online.

SUPPLEMENTARY DATA

## References

[1] Conrad D.F., Pinto D., Redon R., Feuk L., Gokcumen O., Zhang Y., Aerts J., Andrews T.D., Barnes C., Campbell P. (2010). Origins and functional impact of copy number variation in the human genome. Nature.

[2] MacArthur D.G., Balasubramanian S., Frankish A., Huang N., Morris J., Walter K., Jostins L., Habegger L., Pickrell J.K., Montgomery S.B. (2012). A Systematic Survey of Loss-of-Function Variants in Human Protein-Coding Genes. Science.

[3] Huang N., Lee I., Marcotte E.M., Hurles M.E. (2010). Characterising and Predicting Haploinsufficiency in the Human Genome. PLoS Genet..

[4] Khurana E., Fu Y., Chen J., Gerstein M. (2013). Interpretation of Genomic Variants Using a Unified Biological Network Approach. PLoS Comput. Biol..

[5] Ashburner M., Ball C.A., Blake J.A., Botstein D., Butler H., Cherry J.M., Davis A.P., Dolinski K., Dwight S.S., Eppig J.T. (2000). Gene Ontology: tool for the unification of biology. Nat. Genet..

[6] Petrovski S., Wang Q., Heinzen E.L., Allen A.S., Goldstein D.B. (2013). Genic Intolerance to Functional Variation and the Interpretation of Personal Genomes. PLoS Genet..

[7] Dang V.T., Kassahn K.S., Marcos A.E., Ragan M.A. (2008). Identification of human haploinsufficient genes and their genomic proximity to segmental duplications. Eur. J. Hum. Genet..

[8] Shaikh T.H., Haldeman-Englert C., Geiger E.A., Ponting C.P., Webber C. (2011). Genes and biological processes commonly disrupted in rare and heterogeneous developmental delay syndromes. Hum. Mol. Genet..

[9] Seidman J.G., Seidman C. (2002). Transcription factor haploinsufficiency: when half a loaf is not enough. J. Clin. Invest..

[10] Su A.I., Wiltshire T., Batalov S., Lapp H., Ching K.A., Block D., Zhang J., Soden R., Hayakawa M., Kreiman G. (2004). A gene atlas of the mouse and human protein-encoding transcriptomes. Proc. Natl. Acad. Sci. U.S.A..

[11] Georgi B., Voight B.F., Bućan M. (2013). From mouse to human: evolutionary genomics analysis of human orthologs of essential genes. PLoS Genet.

[12] Iossifov I., Ronemus M., Levy D., Wang Z., Hakker I., Rosenbaum J., Yamrom B., Lee Y.-h., Narzisi G., Leotta A. (2012). De novo gene disruptions in children on the autistic spectrum. Neuron.

[13] Sanders S.J., Murtha M.T., Gupta A.R., Murdoch J.D., Raubeson M.J., Willsey A.J., Ercan-Sencicek A.G., DiLullo N.M., Parikshak N.N., Stein J.L. (2012). De novo mutations revealed by whole-exome sequencing are strongly associated with autism. Nature.

[14] Neale B.M., Kou Y., Liu L., Ma'ayan A., Samocha K.E., Sabo A., Lin C.-F., Stevens C., Wang L.-S., Makarov V. (2012). Patterns and rates of exonic de novo mutations in autism spectrum disorders. Nature.

[15] O’Roak B.J., Vives L., Girirajan S., Karakoc E., Krumm N., Coe B.P., Levy R., Ko A., Lee C., Smith J.D. (2012). Sporadic autism exomes reveal a highly interconnected protein network of de novo mutations. Nature.

[16] De Rubeis S., He X., Goldberg A.P., Poultney C.S., Samocha K., Cicek A.E., Kou Y., Liu L., Fromer M., Walker S. (2014). Synaptic, transcriptional and chromatin genes disrupted in autism. Nature.

[17] Gillis J., Ballouz S., Pavlidis P. (2014). Bias tradeoffs in the creation and analysis of protein–protein interaction networks. J. Proteomics.

[18] Obayashi T., Okamura Y., Ito S., Tadaka S., Motoike I.N., Kinoshita K. (2013). COXPRESdb: a database of comparative gene coexpression networks of eleven species for mammals. Nucleic Acids Res..

[19] Lonsdale J., Thomas J., Salvatore M., Phillips R., Lo E., Shad S., Hasz R., Walters G., Garcia F., Young N. (2013). The Genotype-Tissue Expression (GTEx) project. Nat. Genet..

[20] Ronemus M., Iossifov I., Levy D., Wigler M. (2014). The role of de novo mutations in the genetics of autism spectrum disorders. Nat. Rev. Genet..

[21] Rolland T., Taşan M., Charloteaux B., Pevzner S.J., Zhong Q., Sahni N., Yi S., Lemmens I., Fontanillo C., Mosca R. A proteome-scale map of the human interactome network. Cell.

[22] Robinson P.N., Webber C. (2014). Phenotype Ontologies and Cross-Species Analysis for Translational Research. PLoS Genet..

[23] Doetschman T., Wurst W, Kühn R (2009). Gene Knockout Protocols.

[24] Steinberg J., Webber C. The roles of FMRP-regulated genes in autism spectrum disorder: single- and multiple-hit genetic etiologies. Am. J. Hum. Genet..

