# Supplementary material for: Haploinsufficiency predictions without study bias
Source: Nucleic Acids Res. 2015 May 22;43(15):e101. doi: 10.1093/nar/gkv474 (PMC4551909; doi:10.1093/nar/gkv474)
Supplement: SUPPLEMENTARY DATA [file supp_43_15_e101__index.html]

Haploinsufficiency predictions without study bias — SUPPLEMENTARY DATA 

# Haploinsufficiency predictions without study bias

## SUPPLEMENTARY DATA

- SUPPLEMENTARY DATA
- SUPPLEMENTARY DATA
- SUPPLEMENTARY DATA
