# Supplementary material for: Haploinsufficiency predictions without study bias
Source: Nucleic Acids Res. 2015 May 22;43(15):e101. doi: 10.1093/nar/gkv474 (PMC4551909; doi:10.1093/nar/gkv474)
Supplement: SUPPLEMENTARY DATA [file supp_gkv474_nar-03716-met-n-2014-File004.pdf]

Haploinsufficiency predictions without study bias

Authors: Julia Steinberg[1,2,3], Frantisek Honti[1], Stephen Meader[1], Caleb Webber[1,*]

Affiliations:

[1]MRC Functional Genomics Unit, Department of Physiology, Anatomy and Genetics, University of Oxford, Oxford OX1 3QX, United Kingdom; [2]The Wellcome Trust Centre for Human Genetics, University of Oxford, Oxford OX3 7BN, United Kingdom; [3]current address: Wellcome Trust Sanger Institute, Wellcome Trust Genome Campus, Cambridge CB10 1SA, United Kingdom.

*Corresponding author. Caleb.webber@dpag.ox.ac.uk.

Supplementary Data

Methods

Residual Variance Intolerance Score

To consider the relationship between the Residual Variance Intolerance Score (RVIS) and coding-sequence length (CDS), we performed the following randomisations. We considered 15,144 genes with RVIS scores, known CDS and known numbers of variants obtained from the NHLBI exome server (as for the NoVaDs). For these 15,144 genes, we calculated the proportion of common (MAF>0.1%) among all nonsynonymous and synonymous variants. In each randomization, we permuted the proportions of common nonsynonymous variants. The total numbers of nonsynonymous and synonymous variants for each gene were kept constant; the randomised number of common nonsynonymous variants was obtained from rounding the proportion multiplied by the total number of variants. The randomised number of common nonsynonymous variants was then regressed on the total number of variants in the gene. The studentised residuals from the regression yielded the equivalent of the RVIS in the randomised data.

NoVaDs

We examined the how the NoVaDs was affected by the chosen cut-off of MAF>0.1% for common variants. To this end, we considered the alternative with cut-off MAF>1% for common variants (denoted "NoVaDs_1%") and the alternative with cut-off MAF>0.01% for common variants (denoted "NoVaDs_0.01%"). Both the NoVaDs_1% and the NoVaDs_0.01% were highly correlated with the NoVaDs (Spearman ρ=0.69 and ρ=0.62, respectively). We evaluated how well the NoVaDs_1% and NoVaDs_0.01% predicted human disease genes compared to the NoVaDs, following the same procedure as in the comparison of the NoVaDs and the RVIS. We found that the NoVaDs performed significantly better than the NoVaDs_1% and the NoVaDs_0.01% in all comparisons (**Table S9**).

HIS score predictions

The HIS scores presented in the main paper were constructed using a support vector machine (SVM) with linear kernel. We also considered an SVM with radial kernel, using the same approach as in the main paper.
Applying 10-fold cross-validation to the HIS and HS training sets, the radial kernel SVM achieved an AUC of 0.70 (standard deviation 0.02; **Figure S8**). In total, the radial kernel SVM yielded predictions for 20,557 human genes. Again, we found that 100 subsampling runs of HS genes were sufficient, as the Spearman correlation was ρ>0.996 with scores obtained when repeating the process.
The scores obtained with the linear kernel SVM and the radial kernel SVM were highly correlated (Spearman ρ=0.82, $p<10^{-100}$).

We compared the HIS scores obtained from the linear kernel SVM to the radial kernel SVM using the Mann-Whitney rank test and all gene sets as in the main text.

The “radial kernel SVM” score outperformed the “linear kernel SVM” on the “OMIM HI” and “CGD AD” genes using both the MCC and the AUC metric (all $q<10^{-4}$), and on the “OMIM HI *de novo*” and “MGI Lethality” genes using the AUC metric (both $q<10^{-4}$; **Figure S7**, **Tables S7, S8**).

By contrast, across both the MCC and the AUC metrics, the linear kernel SVM significantly out-performed the radial kernel SVM on “MGI Seizures”, “SMP Viability” and “SMP Viability new” genes (all $q<10^{-8}$), as well as on all three autism gene sets (all $q<0.01$).

Notably, the “radial kernel SVM” fitted the training set more strongly than the linear kernel SVM (mean AUC higher by 0.03; Mann-Whitney $p<10^{-10}$), consistent with the significantly better performance for better-studied genes and significantly worse performance for less-studied genes observed here.

These results show that stronger tuning to the training set does not necessarily translate to consistently better performance on other gene sets. In particular, the “gold standard” set of haploinsufficient genes we used to construct the predictions comprises very well-studied genes. The radial kernel SVM, which achieved slightly better performance on this “gold standard”, did not perform consistently better on less-studied genes, and made predictions for significantly fewer genes without Pubmed papers (linear kernel SVM: 23.46%; radial kernel SVM: 19.12%; Fisher’s test $p<10^{-10}$). Intuitively, fitting a predictor to capture known genes and thus corresponding biological process does not make it more likely to successfully predict new genes with different biological mechanisms.

Expected AUC and MCC for the ASD genes

Given that only about 50% of the ASD genes (after removal of the training genes) are expected to be causal, what is the expected MCC and AUC under a best-case scenario? We calculated approximate values as follows.

Given 100 ASD genes, if 50 of them are causal, under the best-case scenario, these would be ranked among the most likely to be haploinsufficient. The remaining 50 genes would be random (for simplicity, disregerding possible bias to enable approximate calculations) and thus fall uniformly across the HIS spectrum. The 100 random “control” genes we compare them to would also fall uniformly across the HIS spectrum.

For the AUC, the 50 causal genes would all or amost all have higher scores than any of the random control genes. The random 50 ASD genes would have higher scores than the control genes about 50% of the time. Hence the AUC as the probability that a given ASD gene has a higher score than a given cotrol gene is (0.5*1+0.5*0.5)=0.75.

For the MCC, the 50 causal ASD genes would all fall among genes with the top 25% HIS score (“TP”). Of the remaining 50 non-causal ASD genes, about 13 would fall among genes with the top 25% HIS score (for the MCC, counted as “FN” since we do not know which genes are causal). Finally, of the 100 control genes, about 25 would fall among the genes with the top 25% HIS

score (“FP”) and 75 would fall outside (“TP”). Using the formula in the **Methods**, this yields an MCC of 0.38.

Supplementary figure legends

Figure S1. **The number of Pubmed papers differs between human genes.** 19,957 genes with at least one Pubmed paper are shown.

Figure S2. **a) Genes in various disease sets tend to have a high number of associated Pubmed papers, with different medians between the sets.** Only genes mapped to at least one Pubmed paper are shown (total: 19,957; OMIM HI: 53; OMIM HI *de novo*: 31; CGD AD: 488; MGI Lethality: 80; MGI Seizures: 35; SMP Viability: 184; SMP Viability new: 113).
b) The number of associated Pubmed papers for disease candidate genes. Only genes mapped to at least one Pubmed paper are shown (total: 19,957; ASD1: 46; ASD2: 44; ASD12: 89).

Figure S3. **a) The Residual Variance Intolerance Score (RVIS) depends on gene coding-sequence length (CDS).** In particular, the absolute value of the RVIS (1) is highly correlated with CDS (see **Results**); moreover, the lowest and highest scores are preferentially attained by the genes with highest CDS. **b) The coding-sequence length of genes does not confound the NoVaDS** defined as $\frac{number\ of\ common\ nonynonymous\ varians}{number\ of\ rare\ nonsynonyous\ variants}$ for each gene (see **Methods**). Lower NoVaDS indicates higher intolerance to gene disruptions. **c) The NoVaDS distinguishes disease genes from random genes as good as or better than the RVIS when using the MCC metric.** Disease genes were taken from Petrovski *et al.* and compared to random sets of human genes matched for CDS (see **Methods**). In all comparisons, the average MCC value is higher for the NoVaDS than for the RVIS. Error bars show standard errors across 100 samplings. **d) The NoVaDS distinguishes disease genes from random genes as good as or better than the RVIS when using the AUC metric**
"OMIM HI": 175 genes annotated as haploinsufficient in OMIM; "OMIM HI *de novo*": 108 OMIM HI genes with *de novo* mutations listed in OMIM; "OMIM DomNeg": 364 genes annotated as dominant negative disease genes in OMIM; "OMIM Recessive": 817 genes annotated as recessive disease genes in OMIM; "OMIM disease": 2131 disease genes from OMIM; "MGI Lethality (P)": 91 genes for which the disruption of an orthologue in mouse yields lethality; "MGI Seizures (P)": 95 genes for which the disruption of an orthologue in mouse yields seizures.

Figure S4. **a) The RVIS depends on coding-sequence length (CDS). b-f) The dependance of the RVIS on CDS is retained when the proportion of rare nonsynonymous variants per gene is randomized.** The darkness of the hexagrams represents the number of points falling into the corresponding area of the plot. After randomization, the equivalent of the RVIS was re-calculated; randomizations were repeated 5 times to obtain panels b-f (see **Supplementary Data**). As for the actual RVIS, the absolute value of the score is highly correlated with CDS (Pearson's r between 0.49 and 0.62); moreover,

the lowest and highest scores are preferentially attained by the longest genes both for the actual RVIS and the equivalent in the randomizations.

Figure S5. **a) Genes in various disease gene sets tend to have high coding-sequence length (CDS). b) Disease candidate genes identified through exome sequencing tend to have high coding-sequence length (CDS).**

Figure S6. Among 18 ASD genes with multiple *de novo* loss-of-function mutations ("ASD_M"), cumulative number of genes ranked in the top percentiles. a) The GHIS includes at least as many ASD_M of genes among any of the top percentiles as the Huang HIS score and the Essentiality score. b) The ASD_M coding-sequence length (CDS) is highly correlated with the RVIS, yielding similar rankings. Due to the gene-length bias, it is difficult to disentangle the effect of *de novo* mutations being more likely in long genes on the RVIS, and no like-for-like comparison of the RVIS to the three other methods was possible in this case.

Figure S7. Comparison of GHIS scores (linear SVM) with scores obtained from a radial SVM.
a) Comparison of scores based on known disease genes and mouse phenotypes using the MCC metric. b) Comparison of scores based on candidate disease genes using the MCC metric. c) Comparison of scores based on known disease genes and mouse phenotypes using the AUC metric. d) Comparison of scores based on candidate disease genes using the AUC metric.
Mann-Whitney *p*-values for the comparisons are listed in **Tables S7, S8**.

Figure S8. **a) AUC curves from 10-fold cross-valiadation for the GHIS (linear SVM). b) AUC curves from 10-fold cross-valiadation for the radial SVM.**
Insets show Spearman correlation of the final score with the individual features.

Supplementary tables

See separate Excel file.
Table S1: 31 adult and 4 foetal tissues used to calculate the foetal-to-adult gene expression ratio (F2A).
Table S2: Study bias - Spearman ρ and *p*-values for Figure 1.
Table S3: GHIS scores.
Table S4: Mann-Whitney *p*- and *q*-values for MCC for Figure 2a,c.
Table S5: Mann-Whitney *p*- and *q*-values for AUC for Figure 2b,d.
Table S6: Mann-Whitney *p*- and *q*-values for MCC and AUC for Figure S3.
Table S7: Mann-Whitney *p*- and *q*-values for MCC for Figure S7a,b.
Table S8: Mann-Whitney *p*- and *q*-values for AUC for Figure S7c,d.

Table S9: Comparison of NoVaDs to NoVaDs_1% and NoVaDs_0.01%

1. Petrovski, S., Wang, Q., Heinzen, E.L., Allen, A.S. and Goldstein, D.B. (2013) Genic Intolerance to Functional Variation and the Interpretation of Personal Genomes. *PLoS Genet*, **9**, e1003709.
