# Supplementary figures and images for: Haploinsufficiency predictions without study bias
Source: Nucleic Acids Res. 2015 May 22;43(15):e101. doi: 10.1093/nar/gkv474 (PMC4551909; doi:10.1093/nar/gkv474)

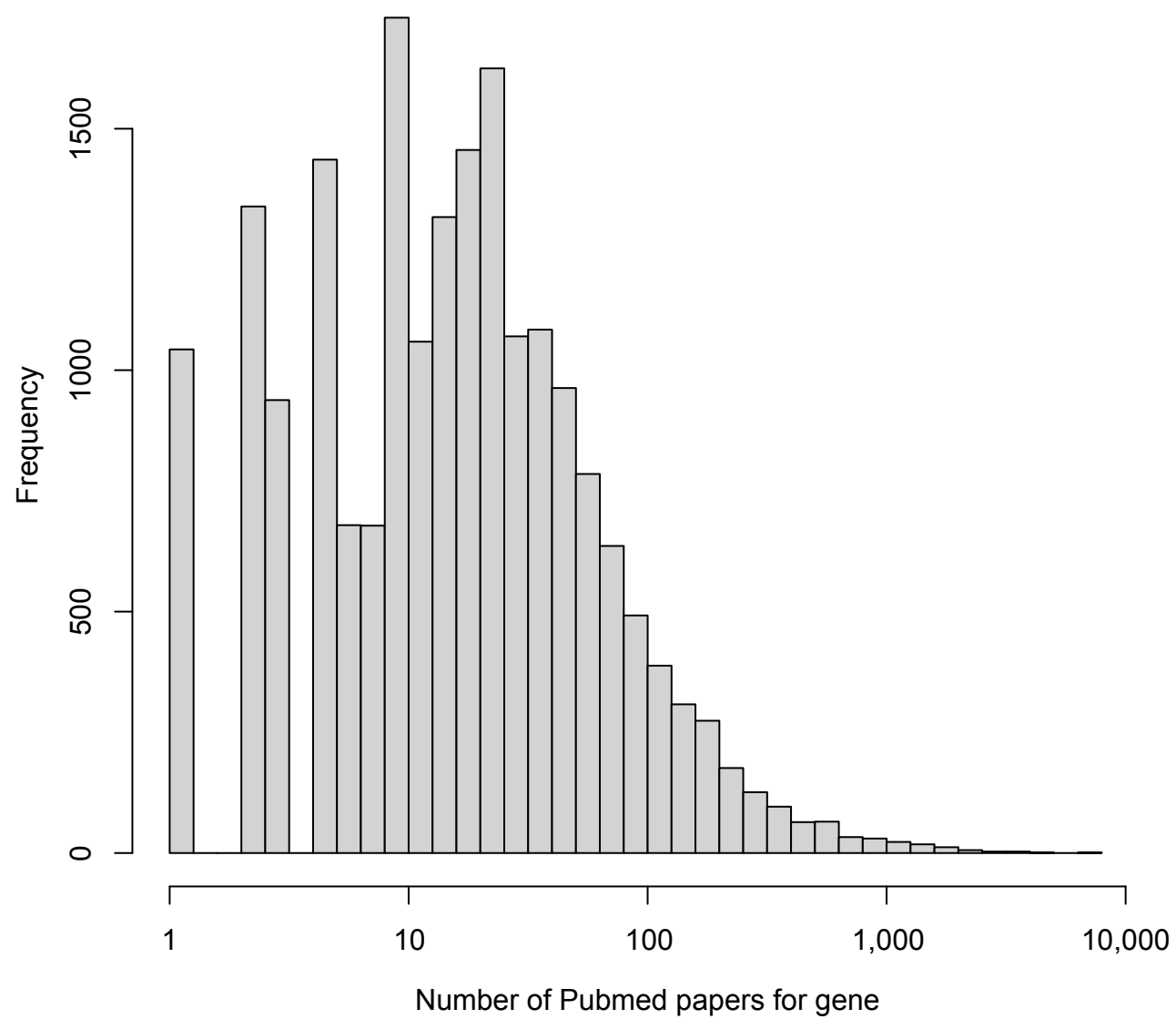

Figure S1

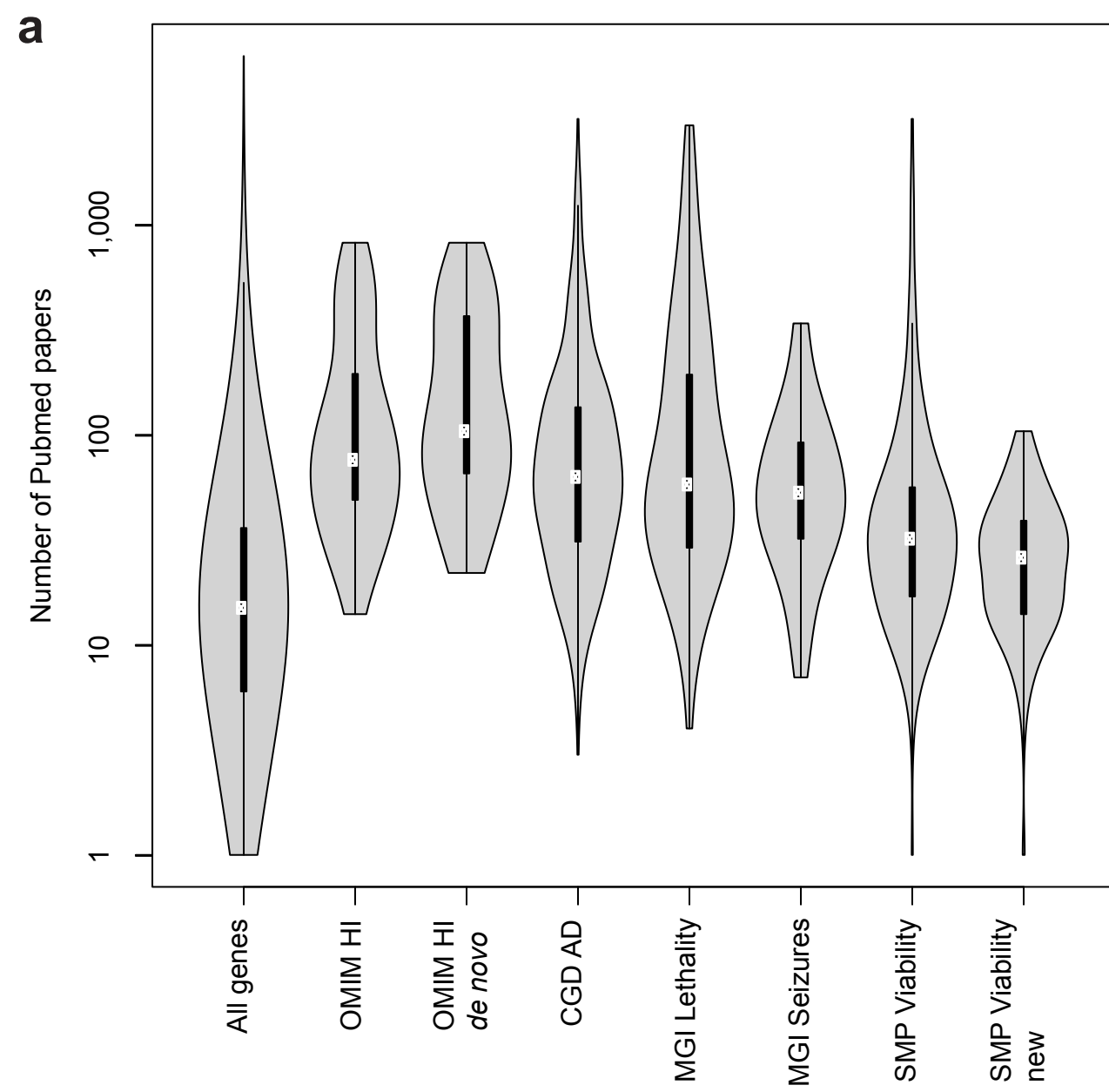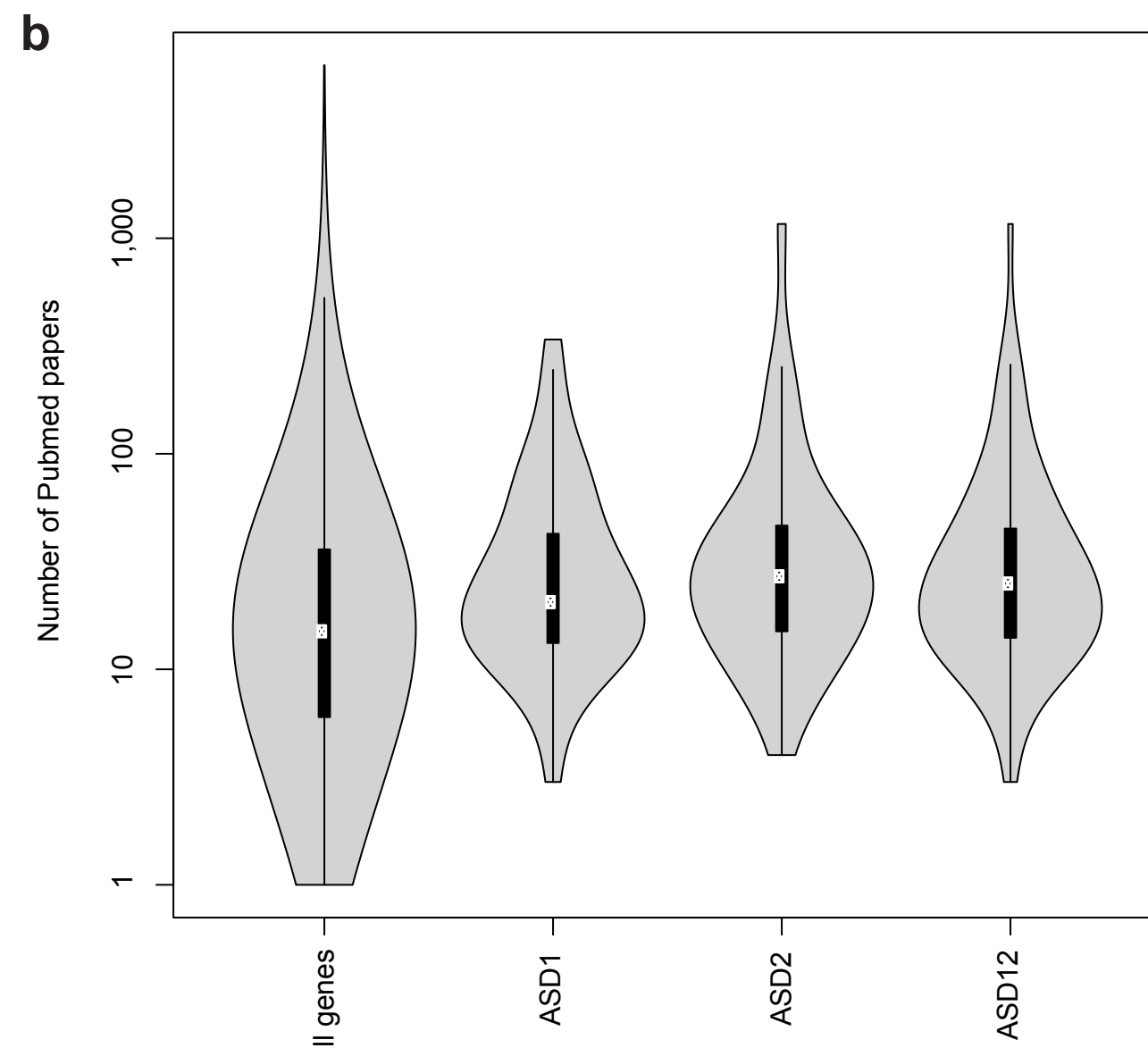

Figure S2

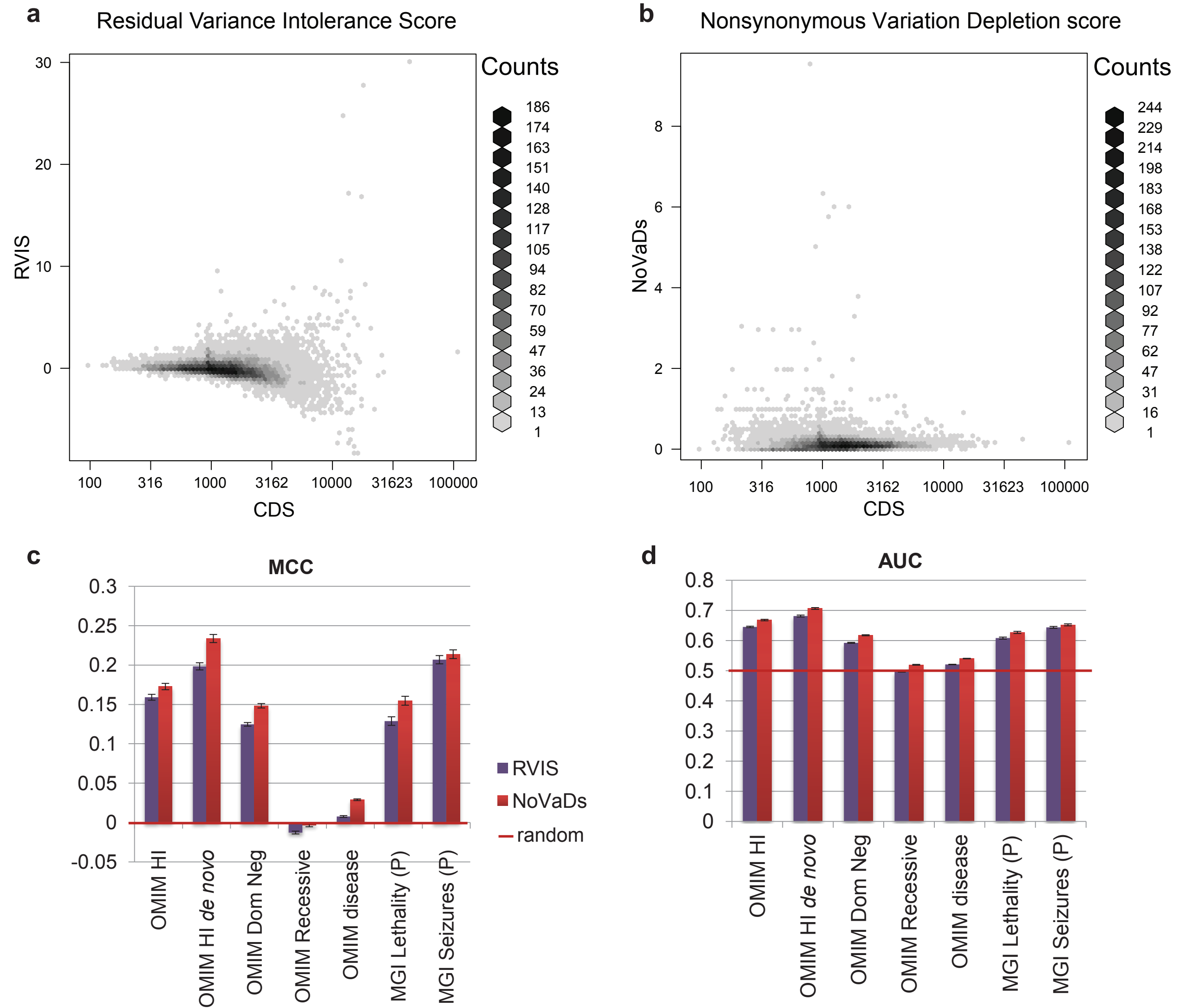

Figure S3

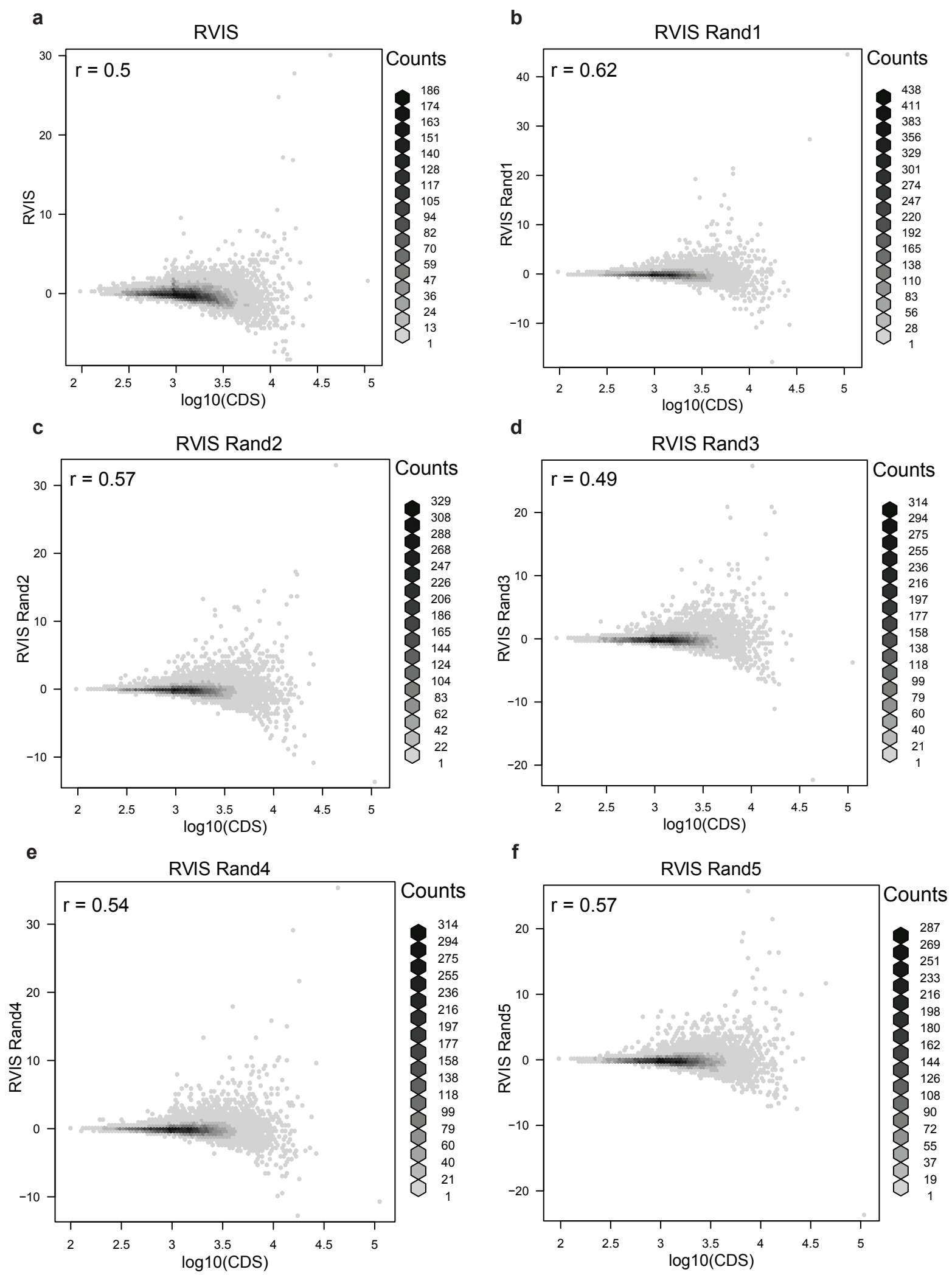

Figure S4

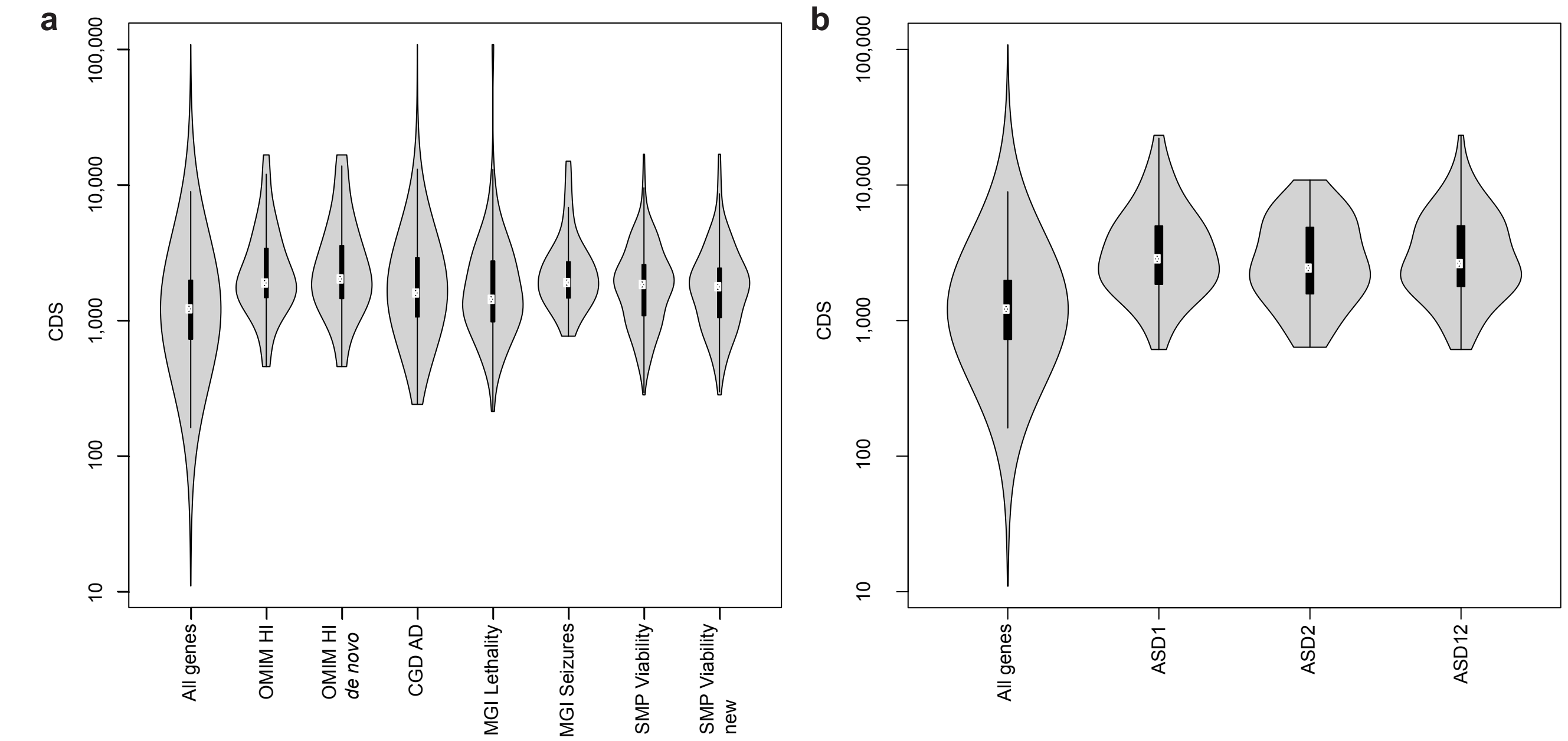

Figure S5

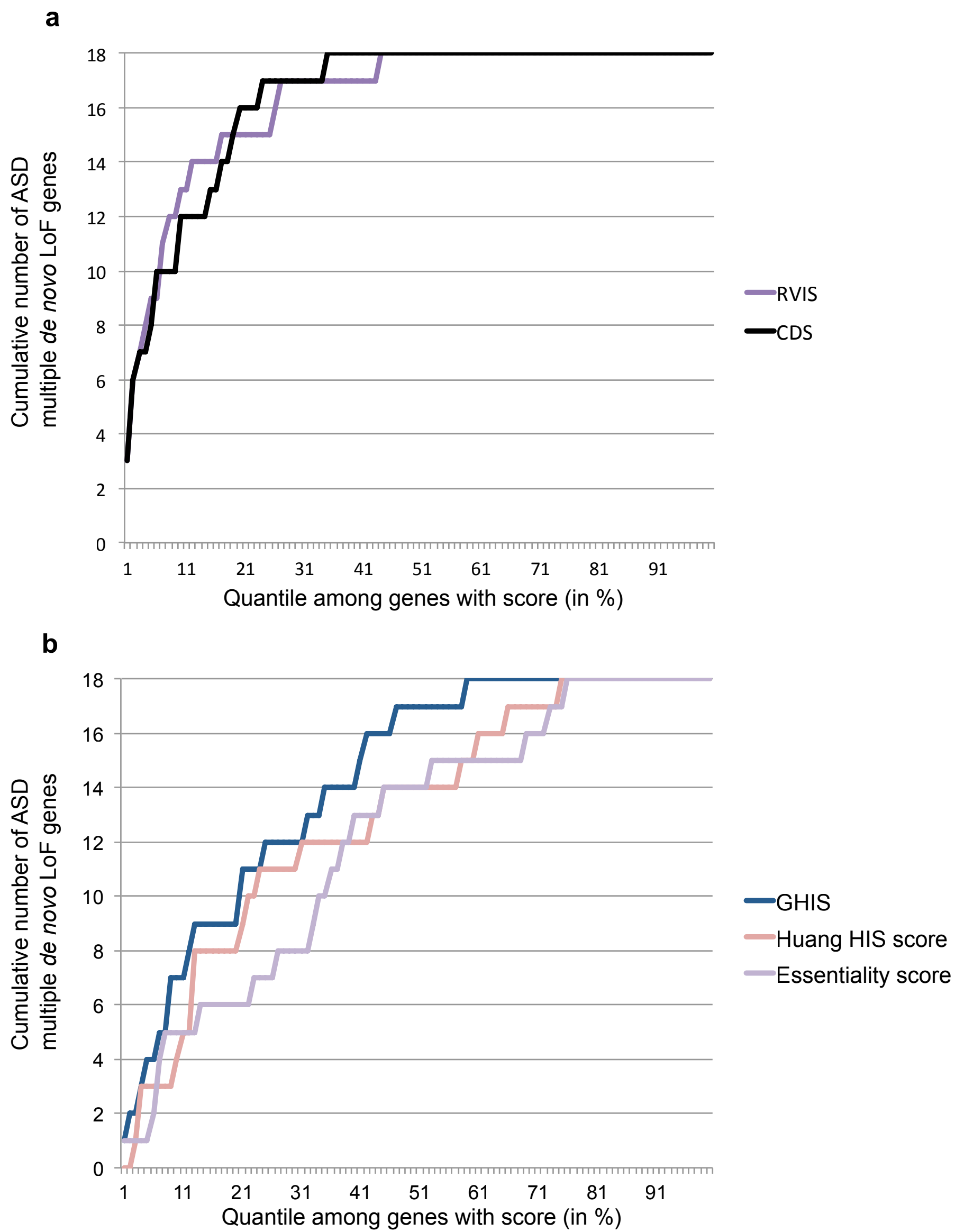

Figure S6

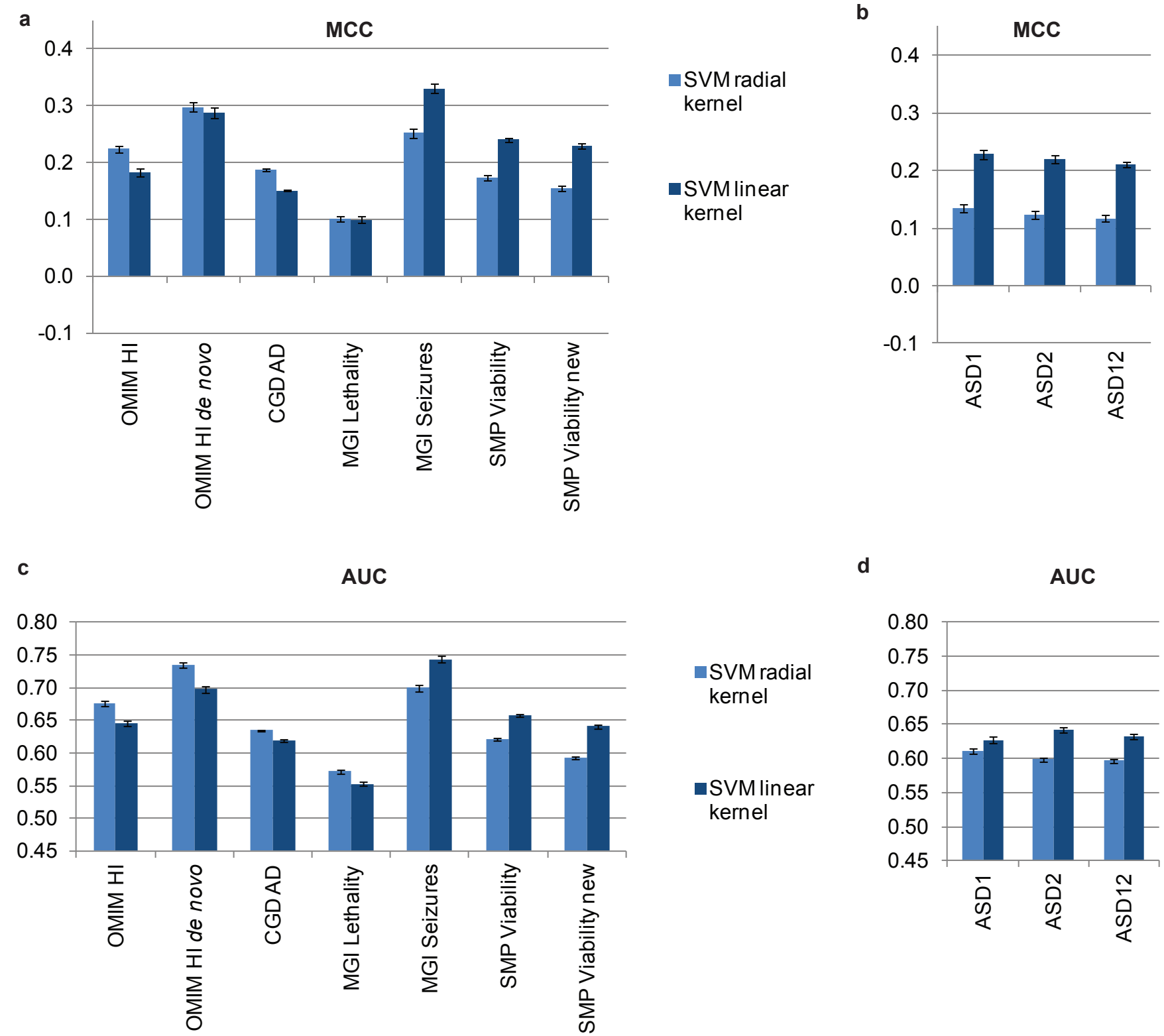

Figure S7

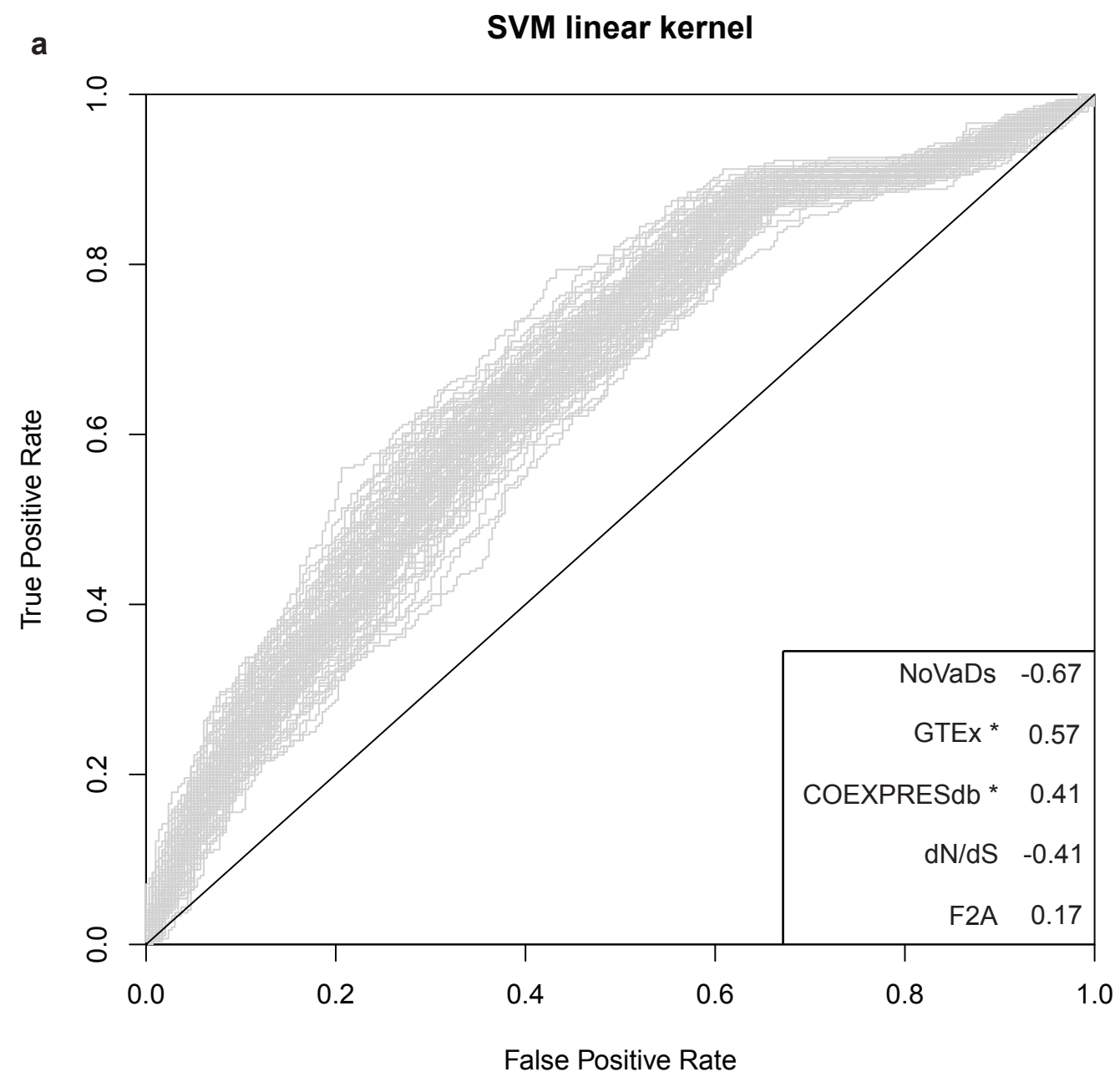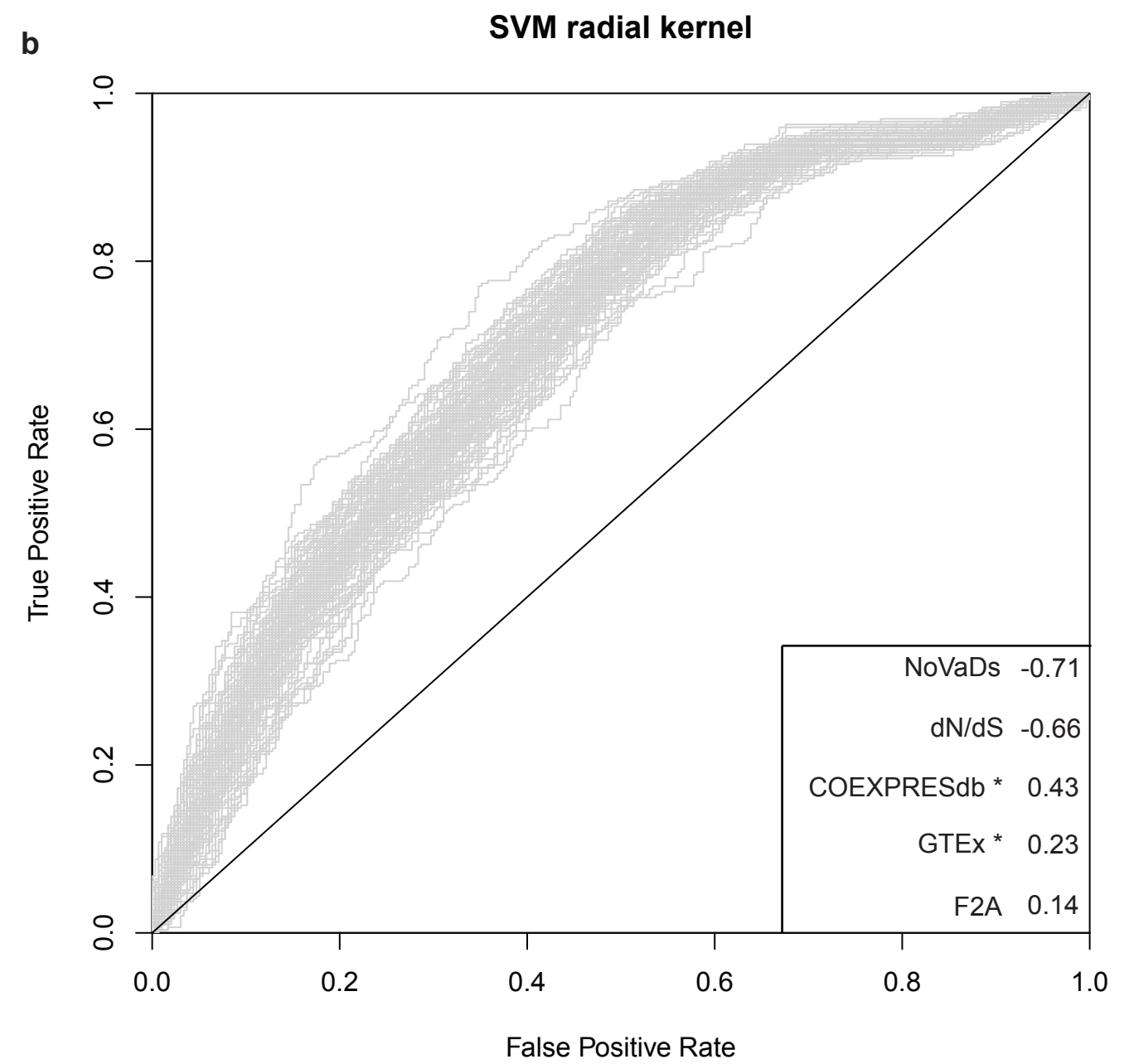

Figure S8

Supplement: SUPPLEMENTARY DATA [file supp_gkv474_nar-03716-met-n-2014-File005.pdf]
